# Chemiluminometric Immunosensor for High-Sensitivity Cardiac Troponin I Employing a Polymerized Enzyme Conjugate as a Tracer

**DOI:** 10.1038/srep14848

**Published:** 2015-10-07

**Authors:** Guei-Sam Lim, Sung-Min Seo, Sung-Ho Paek, Seung-Wan Kim, Jin-Woo Jeon, Dong-Hyung Kim, Il-Hoon Cho, Se-Hwan Paek

**Affiliations:** 1 Department of Bio-Microsystem Technology, Korea University, Sungbuk-gu, Seoul 136-701, Korea; 2 Devices and Materials Laboratory, LG Electronics Advanced Research Institute, Seocho-gu, Seoul 137-724, Korea; 3 Department of Biomedical Laboratory Science, Eulji University, Seongnam, Gyeonggi-do 461-713, Korea; 4 Department of Biotechnology and Bioinformatics, Korea University, Sejong 339-700, Korea

## Abstract

To detect high-sensitivity cardiac troponin I (hs-cTnI; <0.01 ng/mL) at points of care, we developed a rapid immunosensor by using horseradish peroxidase polymerized in 20 molecules on average (Poly-HRP) as a tracer conjugated with streptavidin (SA-Poly-HRP). As shown in the conventional system, enhanced sensitivity could be achieved by using a sequential binding scheme for the complex formation to contain the huge molecular tracer. We used a 2-dimensional chromatographic technology to carry out the sequential bindings in cross-flow directions. After the complex formation of antigen-antibody with analyte in a vertical direction, SA-Poly-HRP was horizontally supplied across the membrane strip for additional binding via a biotin-SA linkage. The HRP substrate was subsequently supplied along the same direction to produce a chemiluminometric signal, which was measured by a cooled charge-coupled device. Hs-cTnI analysis was completed in this format within 25 min, and the results showed a high correlation with those of the CentaurXP® reference system (R^2^ > 0.99). The detection limit of the rapid immunosensor was 0.003 ± 0.001 ng/mL cTnI, corresponding to a 10-fold improvement compared to results using the plain enzyme tracer. This demonstrated the measurement of hs-cTnI in a much more cost-effective manner compared to the automated versions currently available.

The incidence of acute myocardial infarction (AMI) may cause a disorder of the human body or even sudden death^1^. In the United States and Europe, about 15 million people visit the emergency room annually due to their chest pains or other symptoms that suggest AMI. Thus, an accurate diagnosis of AMI based on clinical evidence must be made quickly in order to effectively treat and manage the disease^2^. To this end, the AMI diagnosis is conducted through the results of an electrocardiography (ECG) and cardiac troponin present in the peripheral blood stream, which offer complementary indications in clinical tests^3^. As the ST-segment deviation in an ECG can indicate other conditions, the ECG data alone is not sufficient to accurately diagnose acute coronary syndrome^4^,^5^. This makes it important to explore the central role of cardiac troponin test results in diagnosing AMI^6^.

Cardiac troponins I (cTnI) and T (cTnT) are structural proteins of the cardiac muscle and sensitive, specific biochemical markers of irreversible cellular damage^7^. The protein markers have enabled doctors to identify high-risk patients with acute coronary syndrome. These markers also help to clinically identify patients who are candidates for early coronary angiography or percutaneous coronary intervention^8^,^9^. These markers are more effective than any other markers in clinical AMI diagnosis^10^,^11^. An evidence-based clinical database has rapidly grown for high-sensitivity troponins (for cTnI, hs-cTnI < 0.01 ng/mL^12^). This database has been excellent in diagnosing AMI as early as the first time a patient presents visits with AMI symptoms in the emergency room^13^. As a result, this database may substantially improve the early diagnosis of AMI, particularly in patients with late onset chest pain^14^. Recently, new hs-cTnI or T assays have been introduced to show sensitivity that is higher than those of the conventional assays. In addition, these assays have also improved precision at the lower limit of detection^15^. This outstanding performance was attained through innovation related to assays; specifically, fully-automated versions have been commercially supplied for point-of-care testing (POCT).

As a typical POCT analyzer supporting hs-cTnI measurement, an automated version of enzyme-linked immunosorbent assay (ELISA), PathFast, was developed and launched in the market (refer to Fig. 1^16^). This analyzer captures analytes in four sequential steps including sample injection. The analyzer mixes sample with the captured antibody, carries out the reaction, and washes the unbound analyte based on magnetic separation (1, A). The same four steps are repeated with the detection antibody labeled with an enzyme for the sandwich complex formation (1, B and C). The enzyme substrate is finally added to produce a light signal for detection (1, D). The analyzer covers the clinical dose range of hs-cTnI and allows for a short turn-around time for diagnosis, facilitating fast decision making and patient monitoring^17^. Nevertheless, the total nine steps are sequentially conducted in a costly automated manner, and the signal is detected by photomultiplier tube which is also expensive^18^.

In this study, we substantially simplified the analytical steps to four without sacrificing sensitivity; we employed a two-dimensional (2D) cross-flow chromatography technique used for ELISA-on-a-chip (EOC) as indicated in Fig. 1^19^. In the conventional EOC protocol, the antigen-antibody bindings (1, A and B) and washing (1, C) are serially carried out by capillary action in vertical and horizontal directions, respectively^20^. The washing step has been unique and not shown in other rapid test kits^21^, and can provide a basis to amend the analytical protocol for hs-cTnI via signal amplification. Here, we employed a polymerized enzyme tracer, horseradish peroxidase (HRP) chemically cross-linked in a bunch of 20 molecules on average, in the EOC sensor. Since such a huge tracer can usually cause steric hindrance in the formation of the sandwich binding complex, steps A and B in Fig. 1 were separated. The antigen-antibody bindings without the tracer were induced as usual and the enzyme tracer was subsequently bound via a biotin-streptavidin (SA) linkage by amending the protocol. To this end, the tracing was led either via the second, serial vertical flow or via the first horizontal flow before adding the enzyme substrate solution. To our knowledge, such a 2D chromatography-based assay is the first rapid biosensor format which can be used to measure hs-cTnI even in an emergency and at a much lower cost than when using the existing commercial product, e.g., PathFast analyzer, employing the same ELISA concept.

## Materials and Methods

### Materials

The stock of a cTn I-T-C complex (SRM 2912) and monoclonal antibodies (clones M18, 560, 19C7, and MF4) specific to cTnI were supplied by Hytest (Turku, Finland). Sodium phosphate dibasic, sodium phosphate monobasic monohydrate, sodium chloride, casein (sodium salt form, extract from bovine milk), 3,3′,5,5′-tetramethylbenzidine dihydrochloride (TMB), Sephadex G-15, and D-(+)–trehalose dihydrate were purchased from Sigma (St. Louis, MO, USA). The NC membrane (HiFlowPlus HFB13504) and polyester membrane were supplied by Millipore (Billerica, MA, USA). EZ-Link NHS-LC-LC-Biotin, succinimidyl 4-(N-maleimidomethyl) cyclohexane-1-carboxylate (SMCC), N-succinimidyl 3-(2-pyridyldithio)-propionate (SPDP), dithiotheritol (DTT), goat anti-mouse IgG, and SuperSignal West Femto Chemiluminescent Substrate for HRP were purchased from Thermo Fisher Scientific (Rockford, IL, USA). Streptavidin and HRP were supplied by Calbiochem (San Diego, CA, USA). The cellulose membrane (17CHR, chromatography grade) and the glass fiber membrane for the supply of conjugate were obtained from Whatman (Maidstone, England) and MDI (Ambala Cantt, India) respectively. Hydrogen peroxide, insoluble TMB, and streptavidin-PolyHRP20 (SA-Poly-HRP) conjugate were obtained from Junsei (Tokyo, Japan), Moss (Pasadena, MD, USA), and Fitzgerald (North Acton, MA, USA) respectively. The grade of all reagents was analytical.

### Characterization of the Immunoassay Enzyme Tracers

#### Biotinylation of the detection antibody

The monoclonal antibodies (clones 19C7 and MF4) used for detection were separately coupled with biotin as described in a previous report^22^. Briefly, each antibody in 10 mM phosphate, pH 7.4, containing 140 mM NaCl (PBS) reacted with NHS-LC-LC-Biotin (20 molar excess) dissolved in dimethyl sulfoxide at room temperature for 2 h. The reaction mixture was dialyzed twice in PBS and then concentrated via ultra-filtration. The biotinylated antibody (5 mg/mL concentration) was stored at 4 °C.

### Conjugation of streptavidin with HRP

Streptavidin was coupled with HRP to use the conjugate (SA-HRP) as a signal generator in the conventional immunoassay. Streptavidin (10 mg/mL) in 100 mM phosphate buffer, pH 7.2, (PB) was activated with SMCC in 10 molar excess for 2 h, and the excess reagent was removed on a Sephadex G-15 gel column (10 mL gel volume). HRP (25 mg/mL) reacted in PBS with SPDP in a 20 molar excess for 1 h, and then activated to produce sulfhydryl groups by using 10 mM DTT in 100 mM PB with 5 mM EDTA at 37 °C for 1 h. After separating the excess reagent, the two activated proteins, streptavidin and HRP, were mixed at a ratio of 1:10 and reacted at 4 °C for 9 h. The mixture was combined with the same volume of glycerol and stored at −20 °C until use.

### Microtiter plate-based immunoassay

To characterize the enzyme tracers, we employed a conventional ELISA system utilizing a microtiter plate as the solid matrix^19^. The capture antibody (clone 560) in PBS was immobilized within the microwells (100 μL of 2 μg/mL) by incubating it in a box maintained with 100% humidity at 37 °C for 1 h. After washing it three times with deionized water, the residual surfaces were blocked with PBS containing 0.5% casein (casein-PBS) under the same conditions. After washing it again, the target analyte, cTnI, and the biotinlylated detection antibody (19C7 as single-antibody pair with clone 560; 1 μg/mL) respectively diluted in casein-PBS containing 0.1% Tween-20 (casein-PBS-TW) were sequentially incubated to form sandwich complexes. SA-HRP or SA-Poly-HRP (0.2 μg/mL each) was loaded to react via biotin-SA linkage for 30 min to generate the signal. The HRP substrate solution containing soluble TMB (200 μL) was then reacted for 15 min, and 2 M H_2_SO_4_ solution (50 μL) was added. The optical density of the color produced was measured at an absorbance of 450 nm using a microtiter plate reader (VersaMax, Molecular Devices Inc; Sunnyvale, CA, USA). The identical procedure was repeated with dual-antibody pairs, in which clones 560 and M18 were used as the capture antibodies (1 μg/mL each), and clones 19C7 and MF4 were employed as the detection antibodies (0.5 μg/mL each).

### Construction of the EOC Sensor

#### Preparation of the immuno-strip

An immuno-strip was prepared according to the procedure reported in an earlier study^20^. The strip consisted of four different types of functional membrane pads consecutively connected by partial superimposition. The four membranes were (from the bottom): a polyester pad (4 × 17 mm) for medium application, a glass fiber membrane (4 × 10 mm) as a medium reservoir, an NC membrane (4 × 25 mm) for signal generation, and cellulose chromatography paper (4 × 15 mm) for absorption. The signal generation pad was made by dispensing (1 μL/cm) the capture antibody (clone 560 for single pair, or clones 560 and M18 for double pairs; 1 mg/mL each) and goat anti-mouse IgG (0.1 mg/mL) in PBS onto the respective sites of the NC membrane using a micro-dispenser (a non-contact type; Bio-Dot, XYZ 3000, Irvine, CA, USA). The membrane was dried at 37 °C for 1 h. Finally, the prepared pads were arranged to a width of 4 mm, and each contiguous membrane pad was partially superimposed and fixed onto a plastic film using double-sided tape. A laminating film (3 × 23 mm) was used to cover the surface of the signal generation pad except for the lateral side, which received the horizontal absorption pad (see below)^19^.

### Fabrication of the horizontal absorption pads

Two pads (15 × 13 mm and 11 × 13 mm; 17CHR chromatography paper) were prepared to execute signal generation after antigen-antibody binding. Each pad was used to sequentially supply the enzyme tracer and enzyme substrate solution in a horizontal direction.

### Fabrication of the integrated immunosensor

To provide a functional immunosensing unit, the membrane components were integrated with a plastic cartridge (33 × 76 × 12 mm) consisting of top and bottom plastic plates (refer to Supplementary Figure 1). The bottom plate of the chip consisted of two crossing channels (4 × 61 mm for the vertical channel, 8 × 9 mm square connected to 16 × 2 mm trapezoid for the substrate supply channel, and 16 × 10 mm for the horizontal absorption pad). This was designed to hold the immuno-strip in the vertical direction and to supply the enzyme substrate across the signal generation pad of the immuno-strip in the horizontal direction. The top plate contained a window for signal monitoring and a port (7 × 4 ×4 mm; allowing a maximum sample load of 200 μL) for adding the sample in a vertical direction. The plate also had a port for injecting enzyme substrate and a compartment for the absorption pad in the horizontal arrangement. The substrate injection port (4 × 4 mm) was provided at the far end of the supply channel structure as the inlet of the horizontal flow. The two plates were firmly assembled together using groove joints to retain each component in the respective sites. The assembled immunosensor was stored in a desiccator maintained at room temperature prior to use.

### Optimization and Assessment of EOC Performance

#### Analytical procedures for the colorimetric EOC

The cTnI immunoassay using the EOC was carried out based on a cross-flow chromatographic procedure and colorimetric signal detection. The cTnI stock (1 mg/mL cTnI-C-T complex form) was serially diluted with cTnI-free human serum (determined to be negative for the analyte with the Siemens Centaur XP®). The standard samples were analyzed employing three different procedures: simultaneous reactions for antigen-antibody bindings and enzyme labeling via biotin-SA linkage (Co-reactions), sequential executions of the reactions in the vertical flow (Sequential, Scheme 1), or achievement of the reactions using sequential cross-flows (Scheme 2). The EOC cartridges used for the same or serial experiments were made in a production scale of about 50 replicates or less at the same time. Every three of them were used to test each standard sample under the identical conditions and the signals measured from the replicates were then calculated to determine the mean and standard deviation.

For Co-reactions, each sample (90 μL) was combined with the biotinylated detection antibody (clone 19C7; 10 μL of 3 μg/mL each as an optimal concentration) and, after 5 min, SA-Poly-HRP (e.g., 3 μL of 2 μg/mL) was added. After reactions for the same time period, the mixture was transferred to the application port of the EOC, delivered along the strip by capillary action, and maintained the vertical flow for 15 min. The horizontal absorption pad was placed into the pre-designated compartment and superimposed with the side of the signal generation pad. The HRP substrate containing TMB-M (200 μL) was subsequently added to the injection port and supplied across the signal generation pad of the immuno-strip by capillary action for 5 min. For sequential reactions using Scheme 1, the sample (70 μL) was mixed with the detection antibody (10 μL) and reacted for 5 min. The sample was applied to the EOC and the vertical flow was kept for 15 min. SA-Poly-HRP (e.g., 3 μL of 2 μg/mL) was then added to the conjugation pad and sequentially flown in the same direction by loading casein-PBS for an additional 5 min. The signal was produced as mentioned. For sequential reactions using Scheme 2, each sample (90 μL) was combined with the biotinylated detection antibody (10 μL) and reacted for 5 min. After adding to the EOC and maintaining vertical flow for 15 min, SA-Poly-HRP (100 μL of 0.06 μg/mL) was then transferred to the substrate injection port and maintained to flow across the signal pad for 5 min. After changing the absorption pad to a new pad, the HRP substrate was added to generate a signal.

The color signal produced on the signal pad was captured as an image using a web camera-installed detector and then quantified along the center line of the immuno-strip in a vertical direction using computer software^23^. The optical densities under the analyte peak were normalized against the background present between the signal and control peaks and were then integrated for the area of the immuno-strip between arbitrary position number 131 and 180 so that a numerical signal value could be assigned. The analysis was repeated in triplicate for the same sample, and the mean values for each sample were plotted against the analyte concentration to prepare a standard curve. The detection limit was then determined as three times the standard deviation of the blank value.

### Optimization of analytical components

Using Scheme 2 of the sequential reactions, the concentrations of the two major components, the biotinylated detection antibody and SA-Poly-HRP, were optimized for EOC performance. From this experiment, the dual-antibody pairs were used. By keeping the SA-Poly-HRP constant (0.1 μg/mL), the sample analysis containing 0.1 ng/mL cTnI was first conducted as described in the colorimetric detection procedure at various concentrations of the detection antibody (1–4 μg/mL). The analysis was repeated three times for the same sample, and the mean values were plotted against the detection antibody dose. The assays were repeated under the same conditions except for the absence of the analyte. From the two results, the signal-to-noise ratios were also calculated so that we could plot them. Similarly, the optimal quantity of SA-Poly-HRP was determined to be a range of 0.08 to 0.133 μμg/mL at a constant detection antibody concentration (3 μg/mL).

### Assessment of chemiluminometric EOC performance

The immunoassay procedure using the colorimetric EOC was modified to detect the signal resulting from HRP as chemiluminescence. The analytical procedure was essentially identical to that for colorimetric detection described earlier except for the use of a chemiluminometric substrate solution (100 μL) for the enzyme. Upon completion of the binding reactions with analyte, the substrate solution was added, and the light signal was measured 30 sec after the addition by placing the EOC in a dark chamber installed with a cooled charge-coupled device (cooled CCD; ProgRes MF cool, JENOPTIK, Monheim am Rhein, Germany)^22^. The catalytic light signal was captured as an image using the program provided by the manufacturer and stored in a personal computer. The acquisition time of the cooled CCD camera was fixed to 5 sec as an optimal condition for the signal-to-noise ratio. The image was quantified as optical density and then integrated to determine the signal value. Each analysis was carried out three times for the same sample, and the mean signal values were plotted against the analyte concentration. The detection limit was calculated from the dose-response curve as described earlier. To further determine the uncertainty, the identical procedure was repeated two times and calculated standard deviation for the detection limit.

The dynamic range was also determined from the curve linearized through a logit-log transformation, of which a regression line was obtained using the least-squares method^19^. Logit was calculated as log{B/(B_s_-B)} where B and B_s_ are the signal value for each standard sample and that at saturation, respectively. The saturated chemiluminometric signal was measured by employing a sample containing excess dose of cTnI > 10 ng/mL and the value (the integrated intensity = approximately 10,000) was used to draw the logit-log plot for linearization.

The performance of the chemiluminometric EOC was finally compared with that of a reference system for the same samples. The standard samples were prepared by serially diluting cTnI (stock: 1 mg/mL cTnI-C-T complex) with a cTnI-free human serum. The samples were analyzed using the EOC (as described earlier) and a clinically accepted analyzer, the ADVIA Centaur XP Immunoassay System (Siemens, Erlangen, Germany). The two results for each sample were plotted on the respective axes of a graph to determine the degree of correlation.

## Results and Discussion

### Enzyme Immunoassay Employing Stoichiometric Amplification

#### Introduction of polymerized enzyme tracer

**Figure 2:**
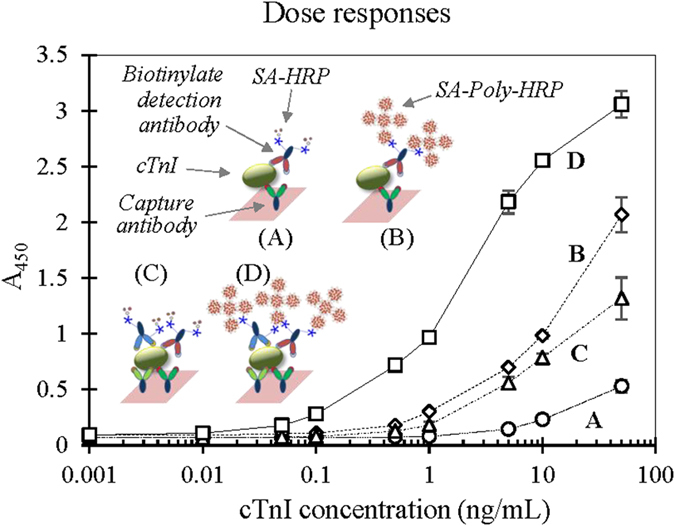
Comparison of dose-response curves of different immunoassay systems for cTnI regarding the signal generator and binding format. The conventional sandwich immunoassay, in which a single pair antibody binding format was used, was first employed for testing the amplification of the signal with various tracers. With the traditional SA-HRP tracer, antigen-antibody bindings occurred in the presence of analyte and a signal proportional to the analyte concentration was subsequently produced (**A**). When SA-Poly-HRP was used, the signal was notably higher than that with SA-HRP over the entire dose range of analyte (**B**). Such signal amplification caused by the stoichiometric increment of the tracer consequently enhanced the detection limit (refer to text for details). (**A**) modified format with dual-antibody pairs showed further enhancement (five times) compared to that obtained with SA-Poly-HRP (**B** vs. **D**), which also resulted in the same effect as the conventional tracer (**A** vs. **C**).

To apply the amplification scheme to an actual immunoassay, we initially used a microtiter plate as the solid matrix for immobilizing the capture antibody. As the microtiter plate format was particularly suitable for carrying out a number of assays at the same time, it was used at the initial stage of this study to screen rate-controlling variables. We first used the conventional antibody sandwich binding system that employs a pair of single capture and detection binders (one-to-one binding format; see Fig. 2). In the presence of sample analyte, the sandwich binding complex was formed and normally linked to an enzyme tracer to produce a signal proportional to the analyte concentration. When the traditional SA-HRP tracer, synthesized via cross-linking between the two different proteins, was introduced, the assay provided quantitative results with a sensitivity of 0.9 ng/mL cTnI (Fig. 2A). Such an analytical capability was not sufficient for the early diagnosis of AMI, which demands a 0.1 ng/mL clinical cut-off for the onset of disease^24^. Therefore, we attempted to enhance assay sensitivity by introducing a polymeric enzyme, Poly-HRP, chemically coupled with SA (SA-Poly-HRP) for stoichiometric signal amplification in place of the traditional tracer. This resulted in an approximate 18–fold increase in detection sensitivity (0.05 ng/mL; Fig. 2B). It is notable that an elevated background level was observed compared to that with the traditional tracer. This result indicated that the novel tracer tended to cause non-specific binding proportional to the tracer molecular size^25^.

### Establishment of an experimental binding model

Because local necrosis of myocardial tissue could be a sign of reversible injury or further AMI, a high-sensitivity measure for a specific marker such as hs-cTnI (e.g., cut-off value of 0.04 ng/mL) becomes important^12^. cTnI is a diagnostic and short-term prognostic marker in patients with AMI and also a predictor of the long-term prognosis of patients with stable coronary artery disease. A scheme of dual-paired sandwich binding complex formation was devised to further enhance performance by further increasing signal intensity^26^. To this end, two pairs of monoclonal antibody combinations were adopted by utilizing two antibodies for capture and two biotinylated antibodies for detection. When the traditional SA-HRP conjugate was used as the tracer, the dual-sandwich complex format showed a 7-fold enhancement in detection capability (0.13 ng/mL; Fig. 2C) compared to that for the single sandwich format (Fig. 2A). Upon combining with SA-Poly-HRP, this sensitivity (0.01 ng/mL; Fig. 2D) improved dramatically—up to 90 times—based on a comparison with that of the conventional format (Fig. 2A). In the case of using the poly-enzyme tracer, the enhanced effect of employing the dual-sandwich format remained about five-fold compared to that obtained with the single paired format.

cTnI immunoassay performance may be influenced by many factors such as post-translational modification and phosphorylation of the protein^26^. This biomarker also forms complexes with other molecules including TnC, heparin, human anti-mouse antibodies, and cTnI-specific autoantibodies circulating in the blood^26^. Considering these clinical problems, we searched for an alternative immunoassay format where dual-paired sandwich complexes with cTnI were used. This development could make the assay insensitive to cTnI modifications and interference, and could, consequently, increase reproducibility and signal intensity. Therefore, dual-antibody pairs were eventually used in the construction of the EOC sensor of this study.

### Development of a Rapid Testing Version of the Enzyme Immunoassay

#### Amendment of analytical protocol for sequential bindings

To diagnose AMI at points of care, such as an in the emergency room or ambulance, we may need a rapid test kit or biosensor that is usually fabricated by using membranes as the solid matrix with a large surface area (e.g., 100-fold larger than that of microwell). This could induce potentially high non-specific binding of the Poly-HRP conjugate onto the solid surface. Non-specific binding, however, is controllable in the EOC device based on 2D chromatography as it adopts the cross-flow washing step as mentioned earlier^27^. The polymeric conjugate can raise the problem of steric hindrance instead, due to the huge molecular size, in the formation of a sandwich complex because the binding reactions normally occur at the same time in the chromatographic assay. Such a potential limitation has been overcome by amending the conventional analytical protocol to realize the sequential bindings as conducted in the microwell-based ELISA.

**Figure 3:**
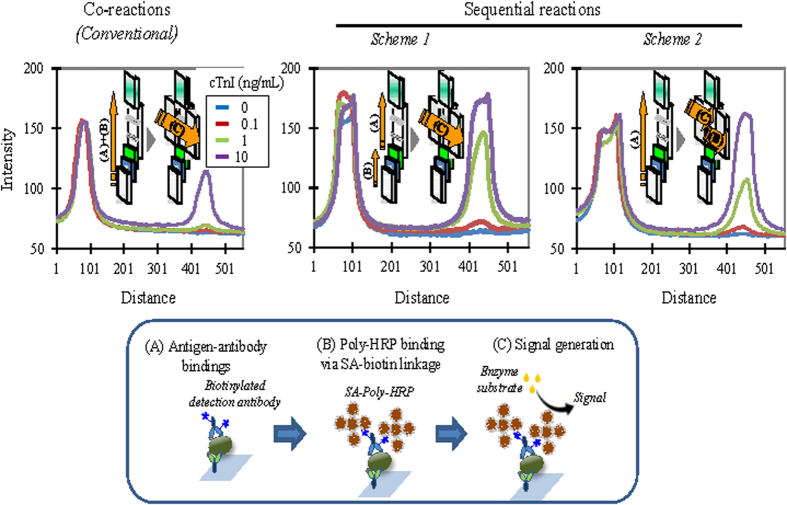
Comparison of typical dose responses yielded according to different analytical protocols for cTnI based on 2D cross-flow chromatography. The sandwich complex formation (process A in the lower panel) and the enzyme binding (**B**) were conventionally carried out in a single step through the vertical flow, and the signal was subsequently generated through the horizontal flow ((**C**) Co-reactions). By using the conventional protocol, 1 ng/mL cTnI could barely be detected in the sample. The detection capability was enhanced by conducting processes A and B in a sequential manner (Sequential reactions). Process B can be carried out either via the second, serial vertical flow (Scheme 1) or via the first horizontal flow prior to the addition of the enzyme substrate solution (Scheme 2). Such amendments of the protocol resulted in an increased detection capability of about 10-fold.

The assay has been conventionally accomplished by concurrently carrying out both the sandwich complex formation (Fig. 3, process A in the lower panel) and the enzyme binding via biotin-SA linkage (process B) through the vertical flow^20^. In this experiment, single-paired antibodies were used to compare the performance obtained according to different analytical protocols. The signal was subsequently generated by adding the enzyme substrate across the signal pad, i.e., via the horizontal flow (process C), and a color signal accumulated for five minutes was produced for easy visualization. The image was captured by a detector and then digitized to optical density using a computer program. As expected, by using the conventional protocol, 1 ng/mL cTnI was barely detectable in the sample due to steric hindrance in the complex formation (3, Co-reactions). To enhance the detection capability, the protocol was revised by separating process A from B and sequentially conducting them in the vertical flows (3, Sequential reactions, Scheme 1). The signal was produced as usual in the horizontal flow, which showed an at least 10-fold increase in sensitivity. Alternatively, the sandwich complex was formed in the vertical flow mode to complete process A, and processes B and C were sequentially carried out in the horizontal flows (Scheme 2; refer to Supplementary Figure 2 for the analytical protocol). The detection capability was about the same as when using Scheme 1 although the signal levels were slightly lowered. To alleviate the steric effect, Scheme 2 was selected as the amended protocol rather than Scheme 1 in this study because of the potential ease of automation.

### Optimization of analytical components

**Figure 4:**
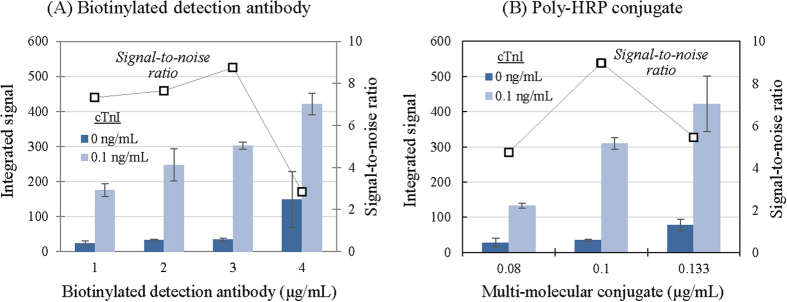
Determination of optimal concentrations of biotinylated detection antibody and SA-Poly-HRP for analytical performance of the colorimetric EOC. The detection antibody in the dual-pair format was increased with other constant components, as the signal for 0.1 μg/mL cTnI in human serum was proportionally augmented, and the background for the absence of analyte was significantly raised only at the highest level in the range (**A**). A similar pattern was obtained when the SA-Poly-HRP quantity was varied (**B**). Optimal concentrations determined based on the signal-to-noise ratio were about 3 μg/mL for the detection antibody and 0.1 μg/mL for the enzyme conjugate. Triplicate tests for each measurement showed variations of 3.3–18.6%.

By using the amended analytical protocol, we then determined the optimal concentrations for the bio-components contributing to stoichiometric signal amplification. In this experiment, the dual-paired antibodies were used (refer to Fig. 2D). Although the analytical time and sample volume are also important parameters determining the assay performances, they are usually maintained minimum for the satisfaction of needs by the market and consumers. These two parameters indeed have been fixed in a previous investigation: e.g., 15 min for the analytical time and <100 μL for the sample volume^19^. In addition, we also determined in an earlier study the optimal conditions for signal generation such as the time and the volume of enzyme substrate solution^20^. With a constant number of capture antibodies, two signal amplification-related components, biotinylated detection antibodies and SA-Poly-HRP, were tested by varying their concentrations (Fig. 4; refer to Supplementary Table 1 for the data). Firstly, when only the detection antibody concentration (as indicated in the figure) was varied, the EOC-based assay for 0.1 n/mL cTnI showed that the signal increased as the antibody level rose (Fig. 4A). The background in the absence of cTnI in the sample was low regardless of the detection antibody amount used (up to 3 μg/mL), but then increased abruptly thereafter. The signal divided by the background—signal-to-noise ratio—consequently reached a maximum at about 3 μg/mL detection antibody. Secondly, the SA-Poly-HRP dose was then changed for optimization, also revealing the up-and-down pattern in the signal-to-noise ratio change (4B). This enabled us to determine the optimal concentration of the conjugate as about 0.1 μg/mL.

Although the amended cross-flow chromatography protocol was employed for the cTnI analysis, its ability to control non-specific binding of the polymerized HRP conjugate was limited particularly at the high concentration of the bio-component. This probably resulted from a synergistic effect of two major factors: the large surface area of the membrane and the huge size of the enzyme conjugate. The surface area of a solid matrix for antibody immobilization may be beneficial for promoting antigen-antibody complex formation as mentioned previously^28^. However, this also increases the total amount of non-specifically bound conjugate even though the bound per unit surface area is small. Furthermore, protein binding onto various solid substrates is usually proportional to size and the binding force can be estimated from the adsorption isotherm. As the HRP conjugate employed in this study was about 20 times larger in molecular size than the intact enzyme, adsorption energy would tend to increase substantially.

EOC performance was then tested under optimal conditions via the cTnI assay according to the amended protocol by adopting the polymerized HRP as the signal generator.

### Performance Characterization of the Chemiluminometric EOC

**Figure 5:**
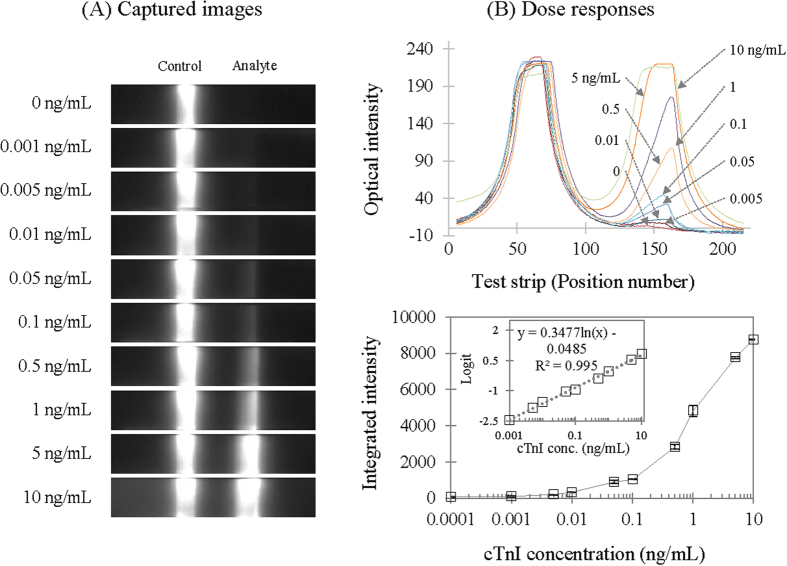
Dose responses of the chemiluminometric EOC sensor to cTnI in the combination with a cooled-CCD as the light detector. Standard samples were prepared with cTnI-free human serum in a range between 0.001 and 10 ng/mL, and were used to test analytical performance. The image captured by the detector (**A**) was converted to optical density, which was then plotted along the vertical location (**B**), the top). The densities under each analyte peak were integrated to allocate the signal value and subsequently graph the value against the cTnI concentration (**B**), the bottom). This dose-response curve showed a typical sigmoidal pattern, of which a linearized form obtained via logit-log transformation offered a dynamic range between 0.005 and 10 ng/mL (inset).

Immunosensor performance was characterized by producing a chemiluminometric signal, which was measured on a detector with a cooled CCD equipped inside^20^. Such a light signal production and detection technique can save time for the total analysis and even enhance the detection capability compared to using a colorimetric EOC (see below). Standard cTnI samples were prepared in a range of 0.001 to 10 ng/mL by spiking into analyte-free human serum and analyzed according to the amended cross-flow chromatography protocol for the EOC (Fig. 5). The signal image was captured 30 seconds after adding the chemiluminometric HRP substrate (Fig. 5A), showing two signal lines: the analyte line immobilized with the dual-capture antibodies and the control with an anti-mouse goat antibody. The signal level on the analyte line appeared proportional to the dose change, whereas the control remained approximately constant as expected. Each image was digitized to obtain the optical density profile along the vertical direction, which was then plotted together with others in an overlaid fashion (Fig. 5B, top). The dose-response curve was drawn by integrating optical densities under each analyte signal curve and by plotting it against cTnI concentration (Fig. 5B, bottom). The intra-assay reproducibility was expressed as the standard deviation at each data point and was listed in Supplementary Table 2. The curve showed a typical sigmoidal pattern and was used to determine the detection limit (0.003 ± 0.001 ng/mL) as the dose corresponding to the integrated signal obtained by multiplying the standard deviation of the background by three^19^. The curve was further linearized via log-logit transformation (Fig. 5B, bottom, inset). As the plot was linear in the dose range between 0.001 and 10 ng/mL, we determined the dynamic range from the detection limit (i.e., 0.003 ng/mL) to 10 ng/mL.

**Figure 6:**
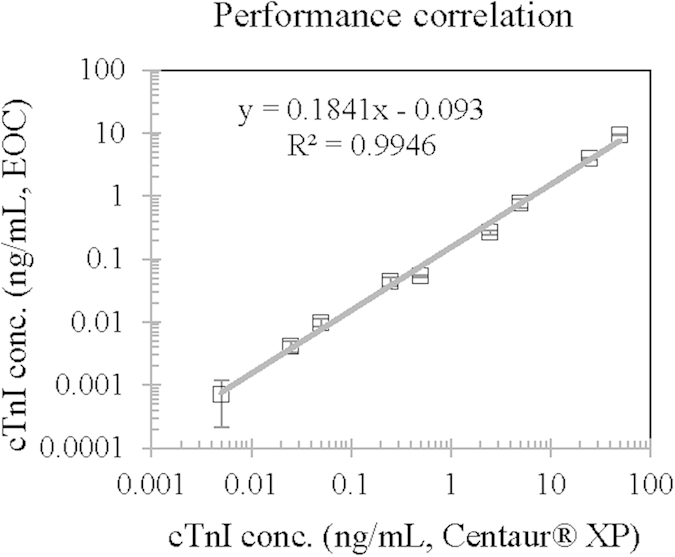
Performance correlation of the chemiluminometric EOC sensor with the reference Siemens CentaurXP® system. Standard cTnI samples prepared in human serum were analyzed using the two systems and the measured concentrations for the same sample were plotted on a graph. The regression line shows a high correlation (R^2^ > 0.99) with the experimental data.

The immunosensor was finally examined to calculate the analytical correlation with a reference device, the ADVIA CentaurXP® Immunoassay System (Siemens). Standard cTnI samples prepared as mentioned above were analyzed using the chemiluminometric EOC and the CentaurXP®, and the respective results for the same sample were plotted on each axis of a graph (Fig. 6; see also the tabulated data in Supplementary Table 3). The figure shows a high correlation (R^2^ > 0.99), indicating that the EOC sensor using the novel polymerized HRP tracer can be used for clinical analysis. However, it seemed that the EOC results were roughly 5 times underestimated for the same samples comparing to those of the reference system. Such difference according to the analyzer used has been reported for cTnI, and standardization for cTnI assay was consequently needed. Universal definition of myocardial infarction recommended at 2009 the use of Standard Reference Material (SRM) 2912 for cTnI. SRM2912 contains the standard material in an intact form of cardiac troponin ICT complexes (>95%)^2^,^29^, which was also used in this study. Indeed, the EOC results showed a 1:1 correlation with those measured by Beckman Coulter Access^19^. On the other hand, some reports of other research groups showed some differences in the determination of cTnI depending on analytical systems used^26^.

The sensitivity of the chemiluminometric sensor (0.003 ng/mL) was about 10 times higher in average than that of a previous EOC (0.027 ng/mL) employing an identical signal processing technique^20^, which used the conventional enzyme conjugate format. Therefore, this enhancement could be attributed mainly to the adoption of the polymeric HRP conjugate as the tracer, which provided stoichiometric amplification. The amended cross-flow chromatography protocol could also be beneficial to enhance the signal-to-noise ratio with the conjugate. Such improvement with the novel tracer was also exemplified with the colorimetry-based sensors and different conjugate formats (0.008 ng/mL for Poly-HRP tracer (refer to Supplementary Figure 3) vs. 0.1 ng/mL for the plain HRP^20^). Chemiluminometry offered approximately three times higher sensitivity than that of colorimetry with the same Poly-HRP, which was consistent with earlier results obtained with the conventional enzyme conjugate (0.027 ng/mL for chemiluminometry vs. 0.1 ng/mL for colorimetry^19^). The relatively short time needed for chemiluminometric detection was another benefit. The chemiluminometric sensor was made of disposable cartridge for one-time use since it can be contaminated after assay with a clinical sample such as whole blood, serum, or plasma. Each cartridge costs about 1.5 US dollars, which however could be reduced in case of mass production.

## Conclusion

The Poly-HRP tracer, which has been used for laboratory immunoassays with a microtiter plate as the solid matrix, was successively incorporated to stoichiometrically amplify the signal into a rapid EOC biosensor based on 2D, cross-flow chromatography. The signal-to-noise ratio representing the sensitivity of the sensor was substantially improved provided the sandwich immuno-complex formation and the enzyme labeling were conducted in the sequential manner. This was achieved by amending the conventional analytical protocol. Furthermore, the chemiluminometric signal generation from the sensor further enhanced the detection capability compared to conventional colorimetric development using the same tracer. Under optimal conditions, hs-cTnI could be measured in clinical samples, facilitating an early diagnosis of AMI at points of care such as in the emergency room or ambulance. As such performance was achieved by using a simple chromatography-based rapid immunosensor, it may be highly competitive with the current commercial products such as PathFast analyzer for the analysis cost of hs-cTnI.

## Additional Information

**How to cite this article**: Lim, G.-S. *et al.* Chemiluminometric Immunosensor for High-Sensitivity Cardiac Troponin I Employing a Polymerized Enzyme Conjugate as a Tracer. *Sci. Rep.* **5**, 14848; doi: 10.1038/srep14848 (2015).

## Supplementary Material

Supplementary Information

## Figures and Tables

**Figure 1:**
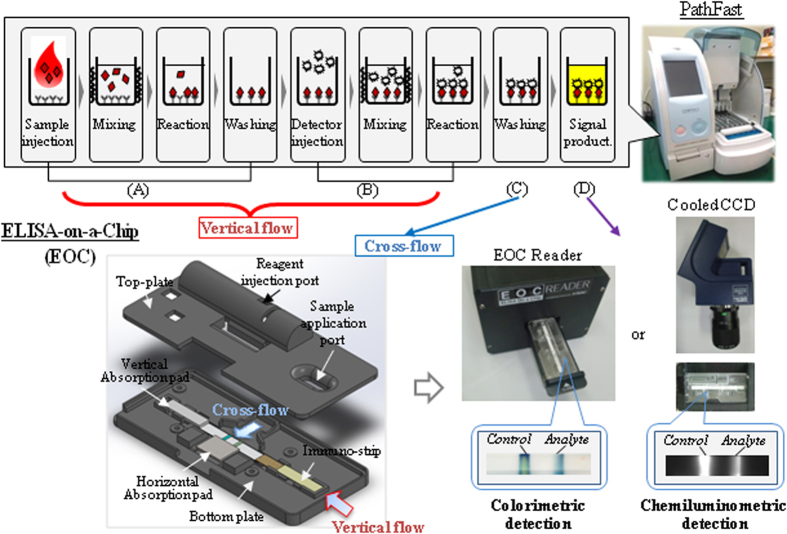
Comparison of the ELISA processes used for a commercial analyzer, PathFast, with those of ELISA-on-a-chip (EOC). The assay procedure on the PathFast analyzer comprises the capture of analyte molecules by the antibody immobilized on magnetic particles (4 steps in (**A**)) and sequential formation of the sandwich complex with the enzyme-labeled antibody (3 steps in (**B**)). After the final washing (**C**), a light signal is produced via the enzyme reaction and then detected using photomultiplier tube (**D**). The nine serial steps are automatically conducted by internally-installed equipment. These, however, can be substantially simplified to three steps as indicated in the use of EOC provided the 2D chromatography technique is adopted. The cross-flow step (**C)** which is currently uniquely used for washing can provide a basis of an amended analytical protocol to amplify the signal (refer to the main text). The cartridge was drawn by Guei-Sam Lim, one of the co-authors.
