# Supplementary material for: Chemiluminometric Immunosensor for High-Sensitivity Cardiac Troponin I Employing a Polymerized Enzyme Conjugate as a Tracer
Source: Sci Rep. 2015 Oct 7;5:14848. doi: 10.1038/srep14848 (PMC4595837; doi:10.1038/srep14848)
Supplement: Supplementary Information [file srep14848-s1.pdf]

# Chemiluminometric Immunosensor for High-Sensitivity Cardiac Troponin I Employing a Polymerized Enzyme Conjugate as a Tracer

Guei-Sam Lim[a,b], Sung-Min Seo[a], Sung-Ho Paek[a], Seung-Wan Kim[a], Jin-Woo Jeon[a], Dong-Hyung Kim[a], Il-Hoon Cho[c], and Se-Hwan Paek[a,d,*]

[a] Department of Bio-Microsystem Technology, Korea University, Sungbuk-gu, Seoul 136-701, Korea

[b] Devices and Materials Laboratory, LG Electronics Advanced Research Institute, Seocho-gu, Seoul 137-724, Korea

[c] Department of Biomedical Laboratory Science, Eulji University, Seongnam, Gyeonggi-do 461-713, Korea

[d] Department of Biotechnology and Bioinformatics, Korea University, Sejong 339-700, Korea

Running Title: Immunosensor for hs-Cardiac Troponin I

[*] Address for correspondence:

Se-Hwan Paek, Professor
540 Biotechnology Building (Green Campus)
Korea University
145 Anam-ro, Seongbuk-gu
Seoul 136-701
Republic of Korea
Tel: 82-2-3290-3438
FAX: 82-2-927-2797

# SUPPLEMENTARY INFORMATION

## Results

### Fabrication of the Integrated Immunosensor

To fabricate a functional immunosensor module, the membrane components were integrated with a plastic cartridge (33 × 76 × 12 mm) consisting of top and bottom plastic plates as shown in Supplementary Figure 1. The bottom plate of the chip consisted of two crossing channels designed to hold the immuno-strip and to supply the signal production-related reagents in the vertical and horizontal directions, respectively (Supplementary Figure 1A). The top plate contained a window for signal monitoring and a port for adding sample in the vertical direction, and also a port for injecting reagent and a compartment for the absorption pad in the horizontal arrangement. The two plates were firmly assembled together using groove joints to retain each component in the respective sites (1B).

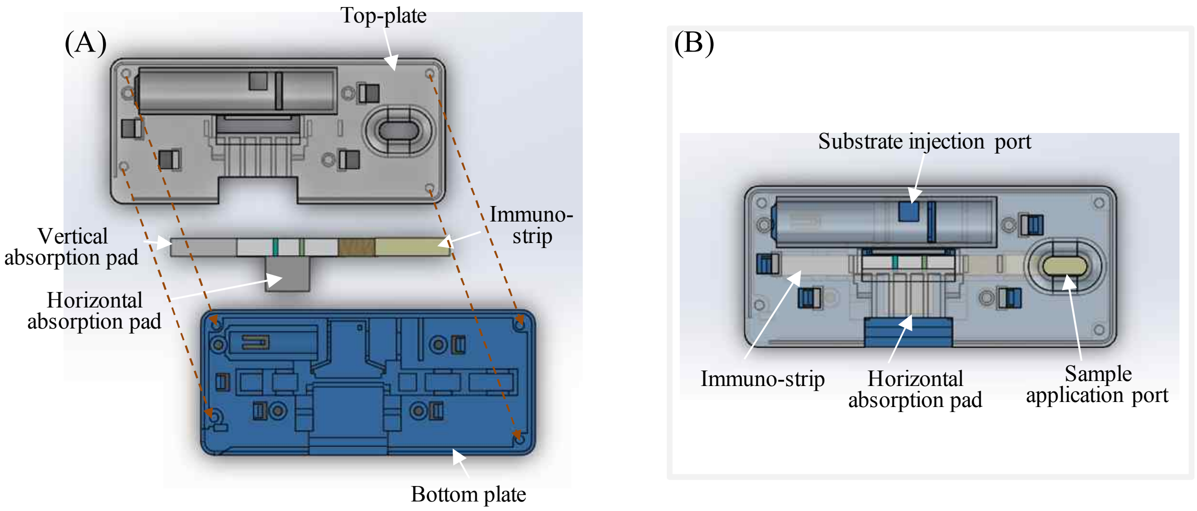


**Supplementary Figure 1.** EOC components (A) and cartridge-type immunosensor after assembly (B). The EOC cartridge was composed of four main components: an immuno-strip, horizontal absorption pads, and top and bottom plastic plates. The membrane components were integrated with the two plates to provide two crossing channels designed to hold the immuno-strip in the vertical direction and to supply the signal production-related reagents in the horizontal direction. After assembly, the cartridge furnished a window for signal monitoring and a port for sample addition in the vertical direction, and also a port for reagent injection and a compartment for the absorption pad in the horizontal arrangement. The

drawings were made by Guei-Sam Lim, one of the co-authors.

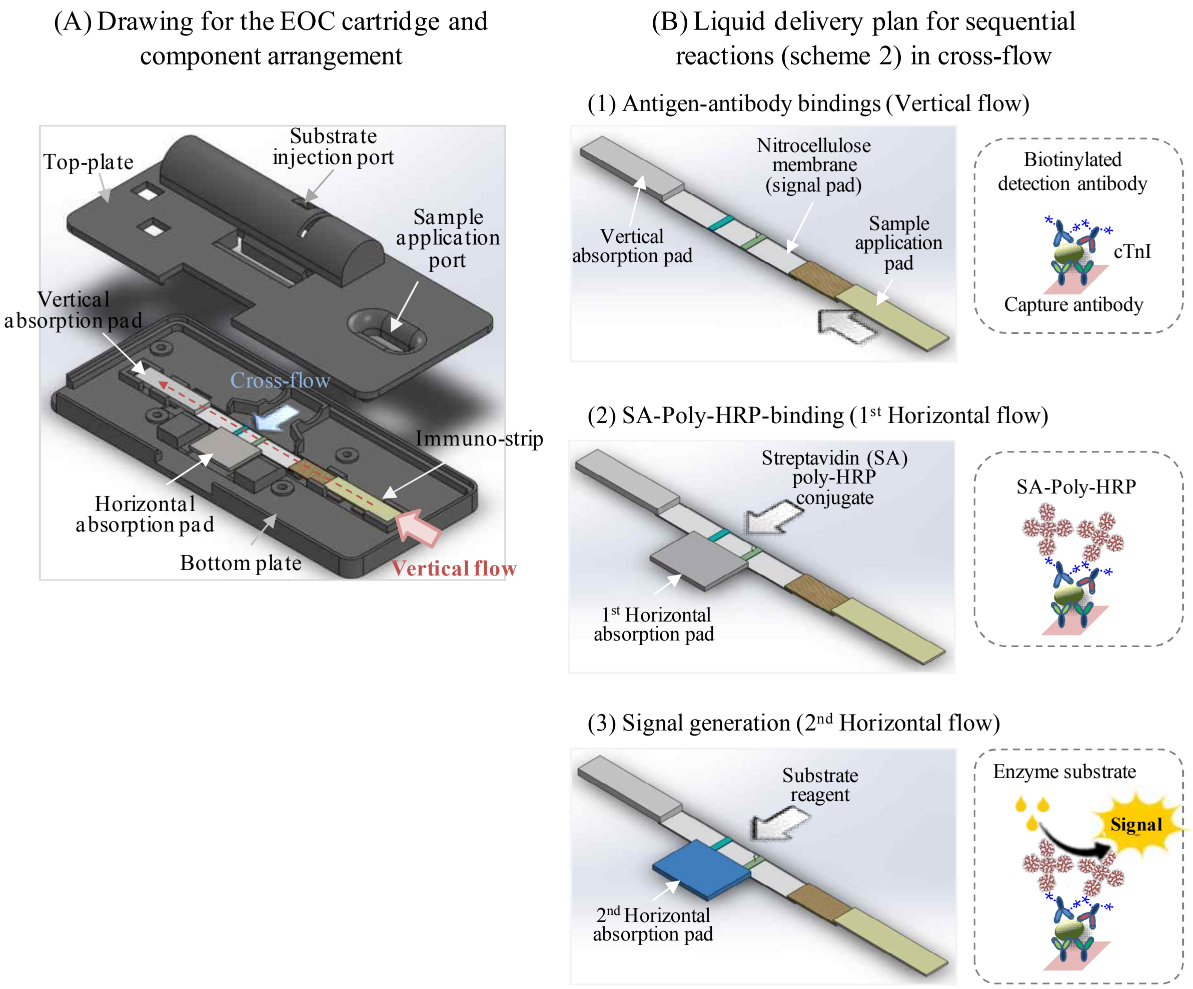


**Supplementary Figure 2.** The analytical concept using EOC based on 2 dimensional, cross-flow chromatography exemplified by sequential reactions in scheme 2 (refer to Figure 3). The immuno-strip placed inside the plastic cartridge (A) drove the sample added by capillary action along the strip in the vertical direction and antigen-antibody bindings can occur on the signal generation pad (B1). The reaction was maintained for 15 min as the vertical absorption pad was placed on the top of the strip. SA-Poly-HRP was then added in sequential manner into the injection port and the first horizontal absorption pad was inserted to contact with the lateral side of the signal generation pad of the strip (B2). The absorption by capillary action induced the horizontal flow and continued for 5 min. Finally, after exchange the horizontal absorption pad with the new one, the enzyme substrate was injected to the same port for signal generation from the enzyme (B3). The illustrations were drawn by Guei-Sam Lim as a co-author.

## Performance Characterization of Colorimetric EOC

We examined whether the novel rapid testing version of the sensor can detect the cTnI present in the high sensitivity region, i.e., < 0.01 ng/mL [1] and further investigated a more convenient way for immuno-sensing by generating a chemiluminometric signal. The colorimetric format of the EOC was used to obtain complete dose responses to the analyte in a range of 0.005 to 5.0 ng/mL, spiked in cTnI-free human serum (Supplementary Figure 3). After analysis, the color that appeared on the EOC signal pad was captured as an image (2A), showing two signal lines: the analyte line immobilized with the dual-capture antibodies and the control with an anti-mouse goat antibody. The signal level on the analyte line appeared proportional to the dose change, whereas the control was maintained approximately constant as expected. The analyte signal for 0.01 ng/mL cTnI could be barely seen with the naked eye. Each image was digitized to obtain the optical density profile along the vertical direction, which was then plotted together with others in an overlaid fashion (3B, 1). Since the signal value was changed as the signal was scanned at different positions of the image, the scan pattern was restricted within a certain area (e.g., the black dotted box as shown in 3C). We used the scan conditions fixed as to the length as well as the position (see the red line in figure). The control peak revealed a bi-modal pattern, indicating overproduction of the enzyme product due to use of excess secondary antibody on the line [2]. The plot was magnified to closely examine the dose responses in the lower dose range, showing that the peak measured at 0.01 ng/mL cTnI was sufficiently discernible from that of the zero analyte (3B, 2). The detection limit (0.008 ng/mL) was determined by multiplying the standard deviation of the background by three.

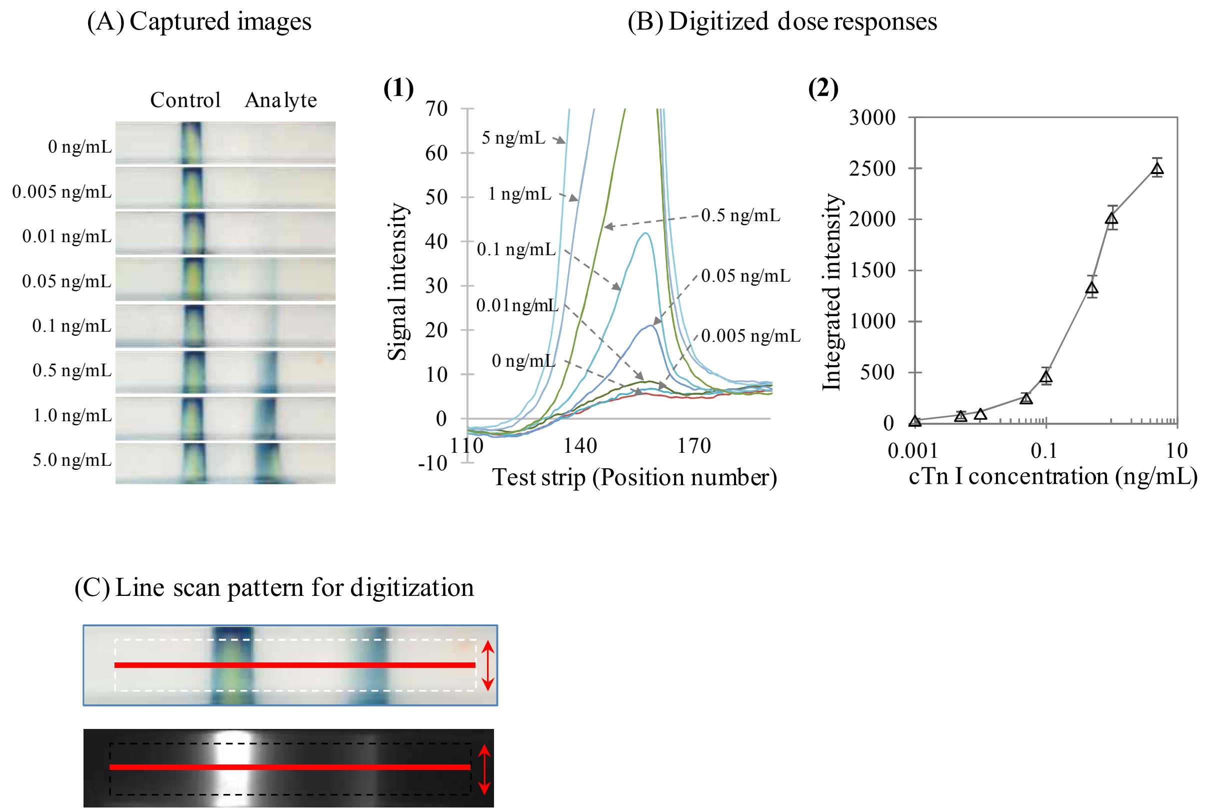


**Supplementary Figure 3.** Dose responses of the colorimetric EOC sensor employing polymerized HRP conjugate as the tracer. Standard samples were prepared by spiking cTnI in analyte-free human serum in a range between 0.005 and 5.0 ng/mL and then analyzed on the EOC sensor. Each signal image was captured using a web camera-installed detector (A) and converted to optical density to plot it against the vertical position of signal pad (B1). The scan conditions for the digitization were fixed as to the length as well as the position (see the red line; C) restricted within a certain area (e.g., the black dotted box). The signal integrated under the analyte curve was plotted against the cTnI dose, showing sensitivity of approximately 0.008 ng/mL cTnI (B2).

**Supplementary Table 1.** Data used for the determination of signal-to-noise ratio in Figure 4.

| Biotinylated detection antibody (µg/mL) | Integrated signal, average | | Standard deviation | | Signal-to-noise ratio |
|---|---|---|---|---|---|
| | 0 ng/mL | 0.1 ng/mL | 0 ng/mL | 0.1 ng/mL | |
| 1 | 23.9 | 175.3 | 6.6 | 18.3 | 7.3 |
| 2 | 32.4 | 247.5 | 3.2 | 45.3 | 7.6 |
| 3 | 34.6 | 302.4 | 3.8 | 10.0 | 8.7 |
| 4 | 148.4 | 421.1 | 80.4 | 30.6 | 2.8 |

| Multi-molecular conjugate (µg/mL) | Integrated signal, average | | Standard deviation | | Signal-to-noise ratio |
|---|---|---|---|---|---|
| | 0 ng/mL | 0.1 ng/mL | 0 ng/mL | 0.1 ng/mL | |
| 0.08 | 28.0 | 132.7 | 12.4 | 7.6 | 4.7 |
| 0.1 | 34.5 | 309.5 | 2.9 | 16.2 | 9.0 |
| 0.133 | 77.4 | 422.4 | 16.6 | 78.4 | 5.5 |

**Supplementary Table 2.** Data used for the dose-response curve in Figure 5.

| cTnI concentration (ng/mL) | Integrated signal, average | Standard deviation | CV% |
|---|---|---|---|
| 0 | 60.3 | 21.9 | 36.42 |
| 0.001 | 93.8 | 19.2 | 20.45 |
| 0.005 | 195.9 | 22.2 | 11.34 |
| 0.01 | 329.2 | 29.6 | 9.00 |
| 0.05 | 894.1 | 98.0 | 10.96 |
| 0.1 | 1031.2 | 21.8 | 2.12 |
| 0.5 | 2835.5 | 132.2 | 4.66 |
| 1 | 4826.4 | 304.9 | 6.32 |
| 5 | 7768.8 | 54.3 | 0.70 |
| 10 | 8765.3 | 4.0 | 0.05 |

**Supplementary Table 3.** Data used for the correlation in Figure 6.

| cTnI (ng/mL) measured by Centaur XP (Reference) | cTnI (ng/mL) measured by EOC | Standard deviation | CV% |
|---|---|---|---|
| 0.005 | 0.0007 | 0.0005 | 69.7 |
| 0.025 | 0.0041 | 0.0008 | 20.6 |
| 0.05 | 0.0098 | 0.0014 | 14.2 |
| 0.25 | 0.0436 | 0.0069 | 15.9 |
| 0.5 | 0.0537 | 0.0017 | 3.1 |
| 2.5 | 0.2633 | 0.0215 | 8.2 |
| 5 | 0.7738 | 0.1236 | 16.0 |
| 25 | 3.9581 | 0.1498 | 3.8 |
| 50 | 9.3973 | 0.0417 | 0.4 |

## References


1. Cho IH, Paek EH, Kim YK, Kim JH, Paek SH (2009) Chemiluminometric enzyme-linked immunosorbent assays (ELISA)-on-a-chip biosensor based on cross-flow chromatography. Analytica Chimica Acta 632 (2):247-255
2. Cho J-H, Paek E-H, Cho I-H, Paek S-H (2005) An Enzyme Immunoanalytical System Based on Sequential Cross-Flow Chromatography. Analytical Chemistry 77 (13):4091-4097. doi:10.1021/ac048270d
